# Supplementary material for: Harnessing strong aromatic conjugation in low-dimensional perovskite heterojunctions for high-performance photovoltaic devices
Source: Nat Commun. 2024 Mar 29;15:2753. doi: 10.1038/s41467-024-47112-y (PMC10980693; doi:10.1038/s41467-024-47112-y)
Supplement: Supplementary file 1 — Supplementary Information [file 41467_2024_47112_MOESM1_ESM.pdf]

Supplementary Information

For

Harnessing strong aromatic conjugation in low-dimensional perovskite heterojunctions for high-performance photovoltaic devices

Bo Li[1,4], Qi Liu[2,4], Jianqiu Gong[1,4], Shuai Li[1,4], Chunlei Zhang[1,4], Danpeng Gao[1], Zhongwei Chen[3], Zhen Li[1], Xin Wu[1], Dan Zhao[1], Zexin Yu[1], Xintong Li[1], Yan Wang[1], Haipeng Lu[3*], Xiao Cheng Zeng[2*], Zonglong Zhu[1*]

[1]Department of Chemistry, City University of Hong Kong, Kowloon, Hong Kong, China

[2]Department of Materials Science & Engineering, City University of Hong Kong, Kowloon, Hong Kong, China

[3]Department of Chemistry, The Hong Kong University of Science and Technology, Clear Water Bay, Kowloon, Hong Kong, China

[4]These authors contributed equally: Bo Li, Qi Liu, Jianqiu Gong, Shuai Li, Chunlei Zhang

* Corresponding author's email: haipenglu@ust.hk, xzeng26@cityu.edu.hk, zonglzhu@cityu.edu.hk,

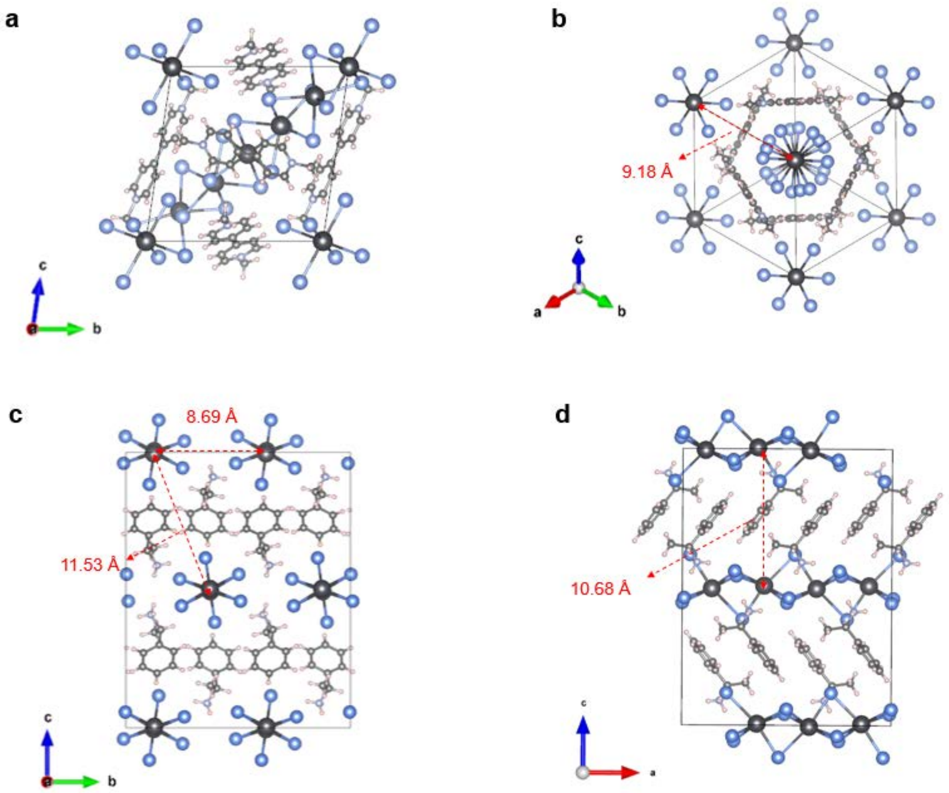

Supplementary Fig. 1 Crystal structure of 1D (**a** and **b**) $MVPb_2I_6$ and (**c** and **d**) $MBAPbI_3$ perovskite.

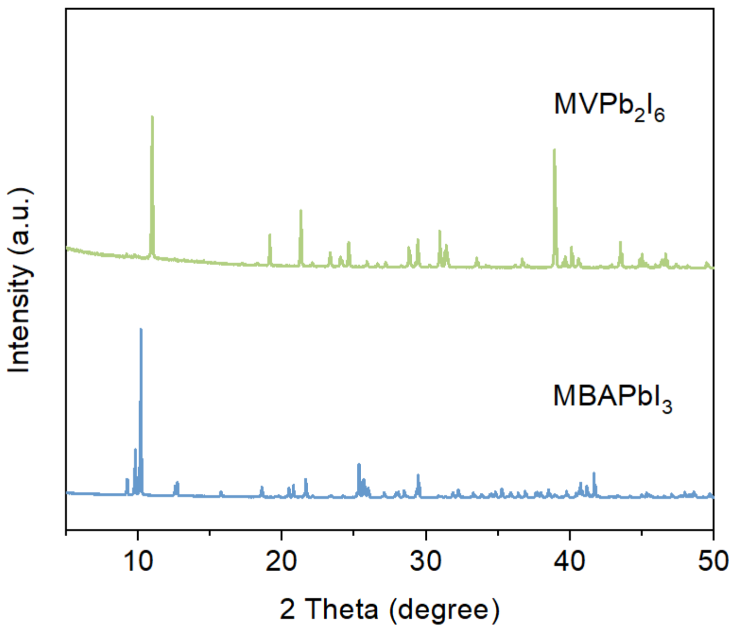

Supplementary Fig. 2 XRD patterns of 1D $MBAPbI_3$ and $MVPb_2I_6$ single crystals.

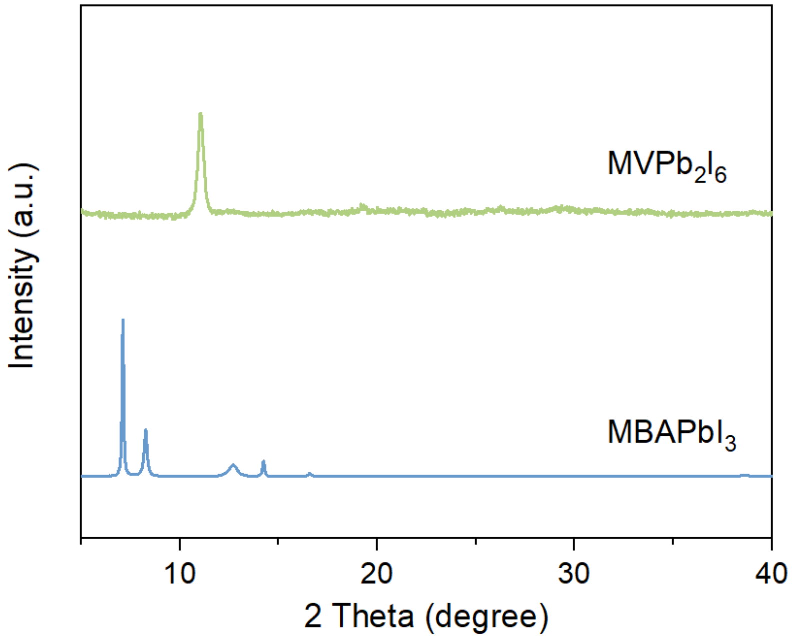

Supplementary Fig. 3 XRD patterns of 1D $MBAPbI_3$ and $MVPb_2I_6$ perovskite films.

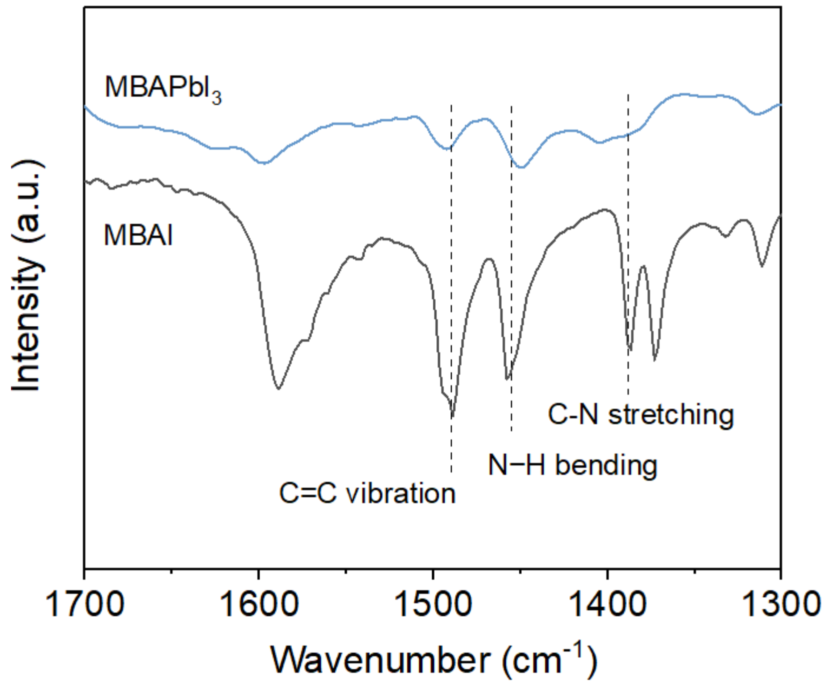

Supplementary Fig. 4 FTIR spectra of MBAI and 1D $MBAPbI_3$.

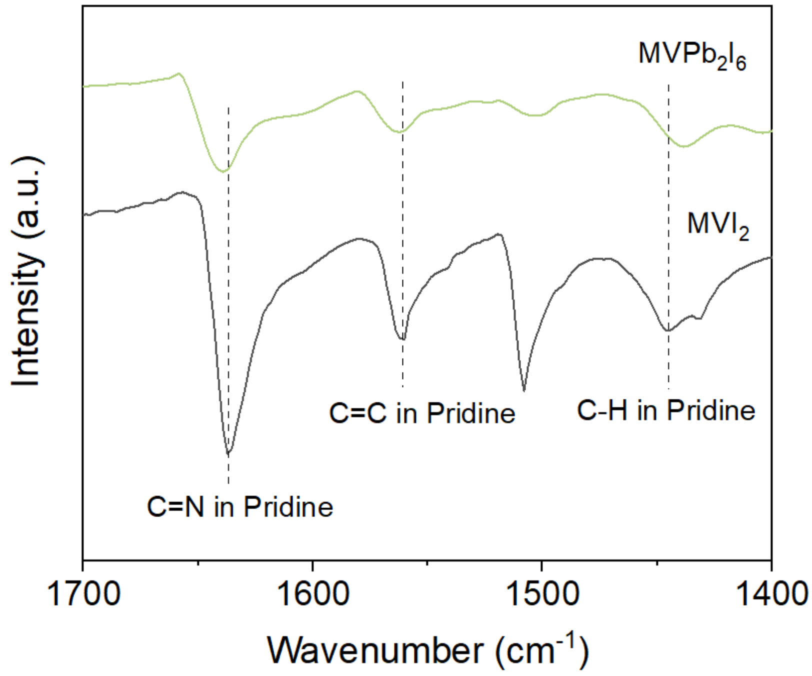

Supplementary Fig. 5 FTIR spectra of MVI_2 and 1D $MVPb_2I_6$.

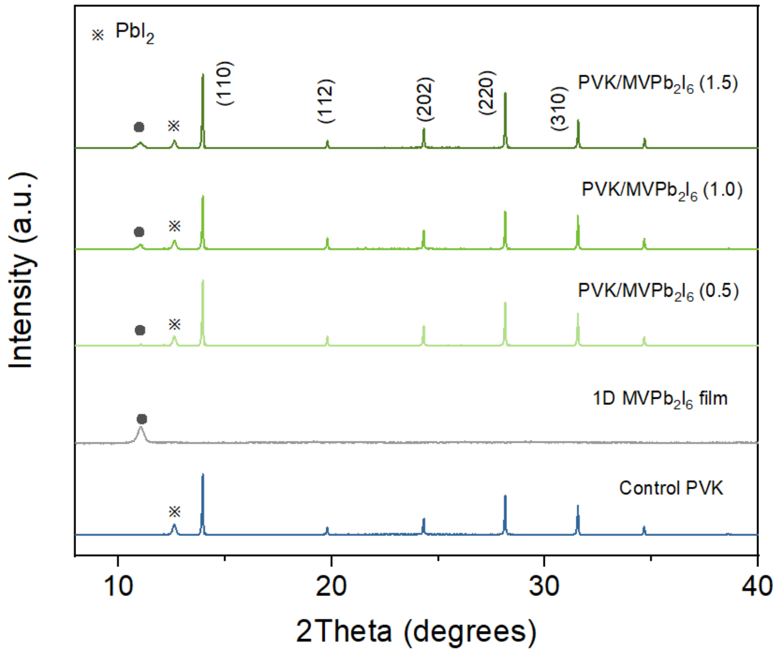

Supplementary Fig. 6 XRD patterns of control perovskite (PVK) and 1D/3D perovskite (PVK) films treated with 0.5, 1.0 and 2.0 mg/mL MVI_2 solution concentrations.

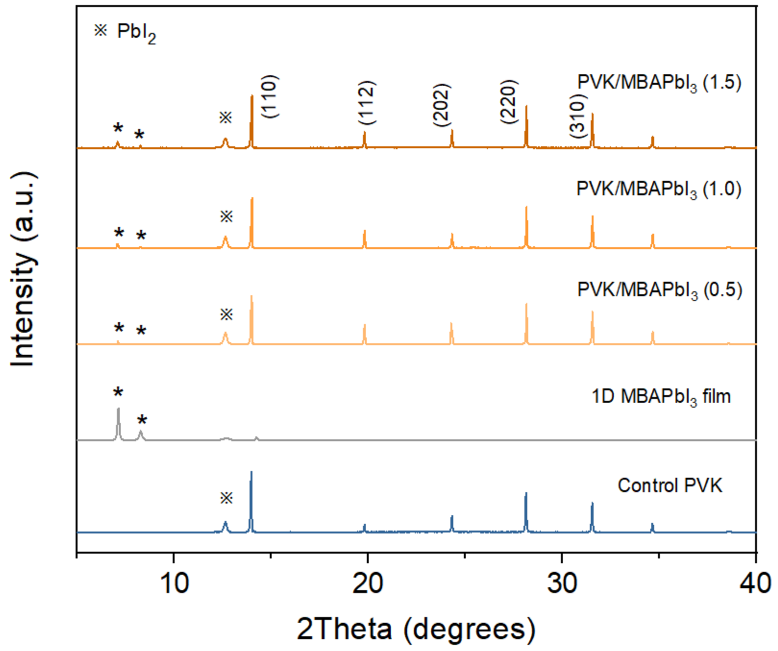

Supplementary Fig. 7 XRD patterns of control perovskite (PVK) and 1D/3D perovskite (PVK) films treated with 0.5, 1.0 and 2.0 mg/mL MBAI solution concentrations.

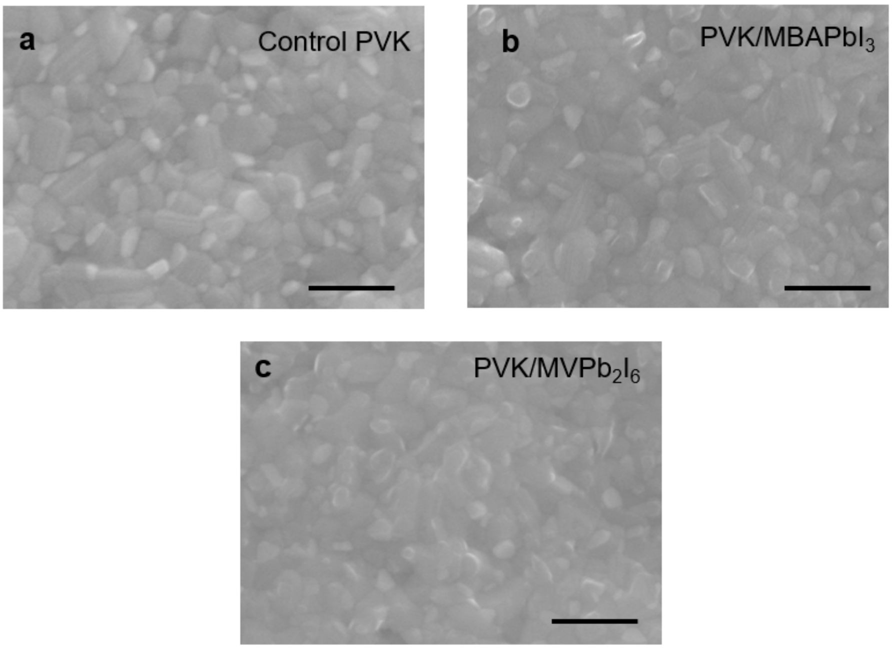

Supplementary Fig. 8 SEM images of (**a**) the control perovskite (PVK) film, (**b**) the perovskite (PVK) / $MBAPbI_3$ film, and (**c**) the perovskite (PVK) / $MVPb_2I_6$ film. The scale bar is 1 μm.

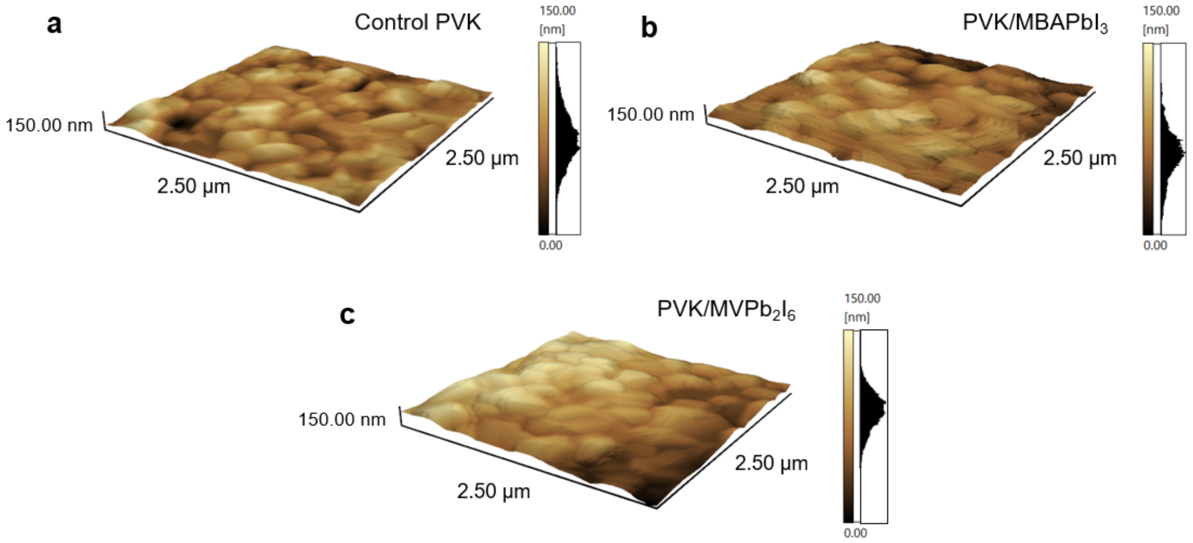

Supplementary Fig. 9 AFM images of (**a**) the control perovskite (PVK) film, (**b**) the perovskite (PVK) / $MBAPbI_3$ film, and (**c**) the perovskite (PVK) / $MVPb_2I_6$ film. The scale bar for surface roughness is 150 nm.

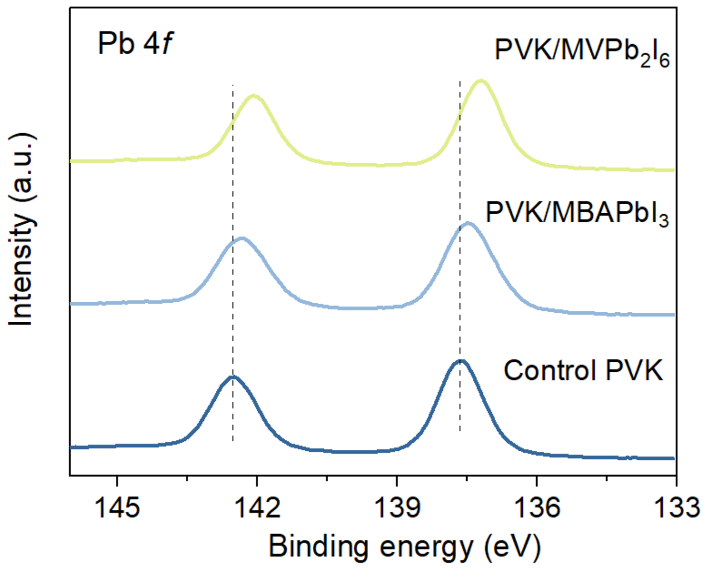

Supplementary Fig. 10 XPS for Pb 4*f* in control perovskite film and LD/3D perovskite films.

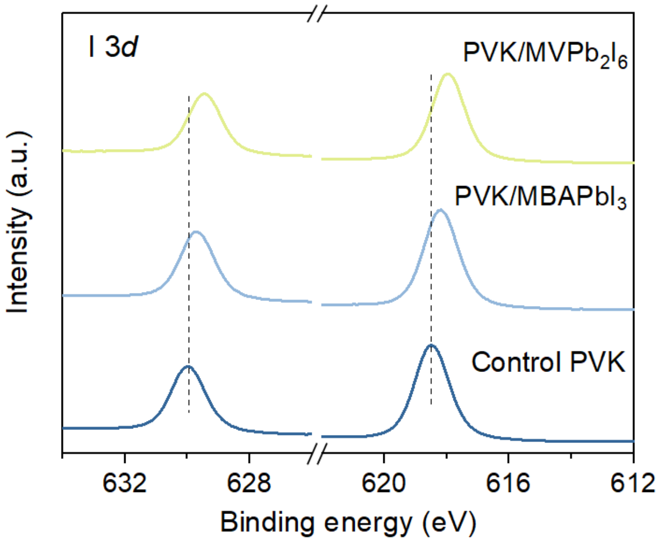

Supplementary Fig. 11 XPS for I 3*d* in control perovskite film and LD/3D perovskite films.

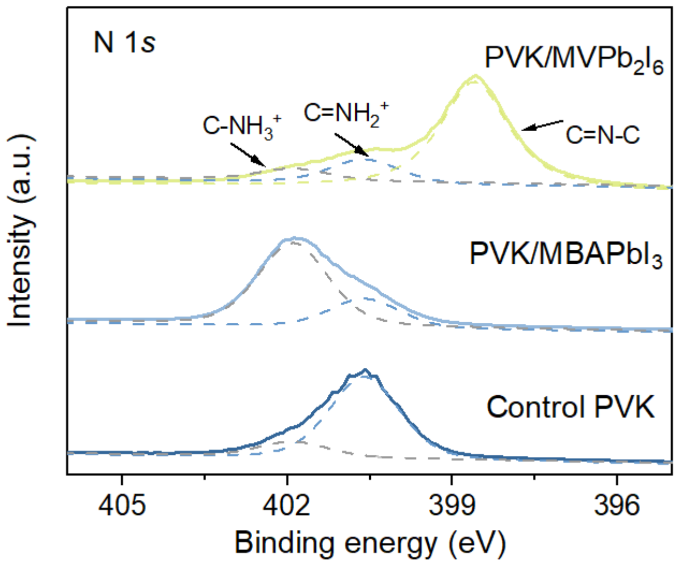

Supplementary Fig. 12 XPS for N 1*s* in control perovskite film and LD/3D perovskite films.

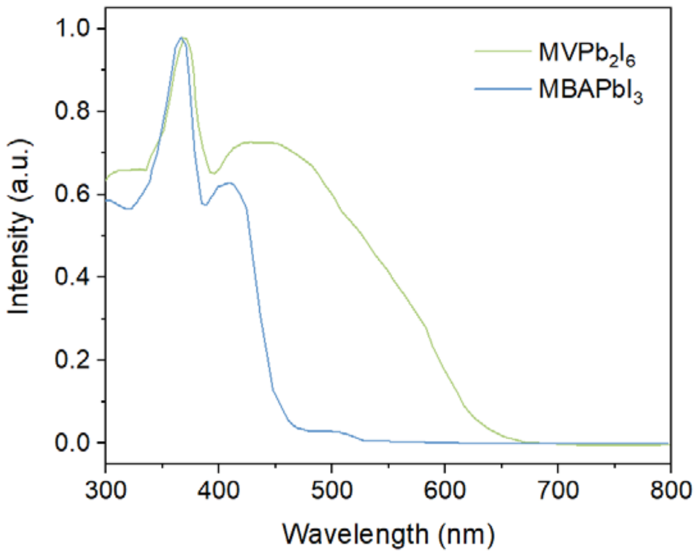

Supplementary Fig. 13 UV-vis spectra of 1D $MBAPbI_3$ and $MVPb_2I_6$ perovskite single crystals

Supplementary Note 1

For Space charge limited current (SCLC) measurements, the electron-only devices in Supplementary Fig. 14 were prepared to calculate the defect densities and carrier mobilities. In the SCLC regime, the current is dominated by charge carriers injected from the contacts and the current-voltage characteristics become quadratic (I~V^2). Supplementary Fig. 14 shows the J-V curves of the fabricated devices on a double logarithmic scale, which comprises the Ohmic region, the trap-filling limit (TFL) region and the Child region. In the TFL region, the trap-state density (N_t) can be calculated by the following equation:

$$N_t = \frac{2\varepsilon\varepsilon_0 V_{\mathrm{TFL}}}{qL^2} \quad .... \text{(S1)}$$

where ε and ε_0 are the relative dielectric constant and vacuum permittivity, respectively. V_{TFL} is the onset voltage of TFL region, q is elementary charge. In the child regime, the carrier mobility is evaluated using the Mott-Gurney law:

$$\mu=\frac{8J_{\mathrm{D}}L^3}{9\varepsilon\varepsilon_0 V^2} \quad .... \text{(S2)}$$

where J_{D} is the current density and L is the crystal thickness.

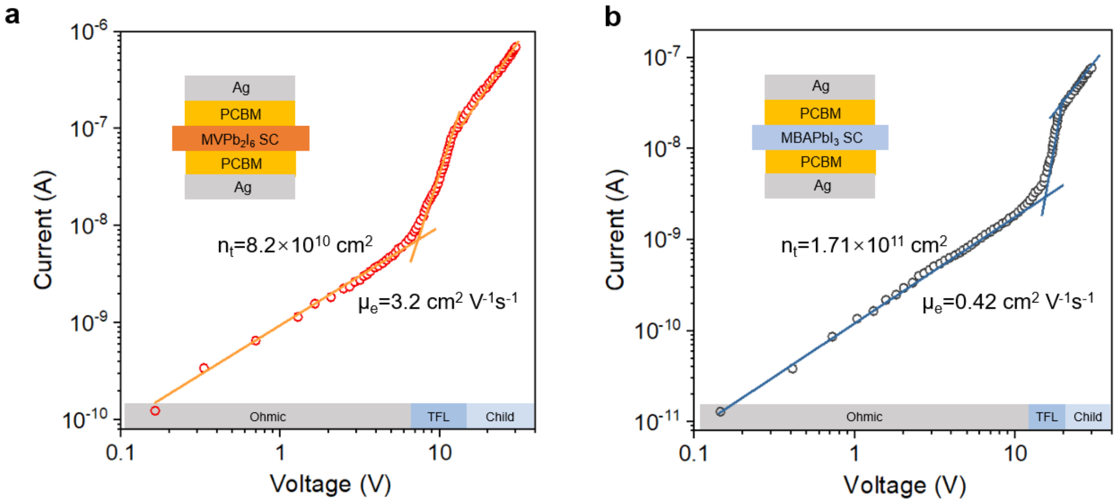

Supplementary Fig. 14 Current-voltage curves of electron devices of (**a**) $MVPb_2I_6$ SCs and (**b**) $MBAPbI_3$ SCs, respectively. Solid lines are obtained by fitting the data.

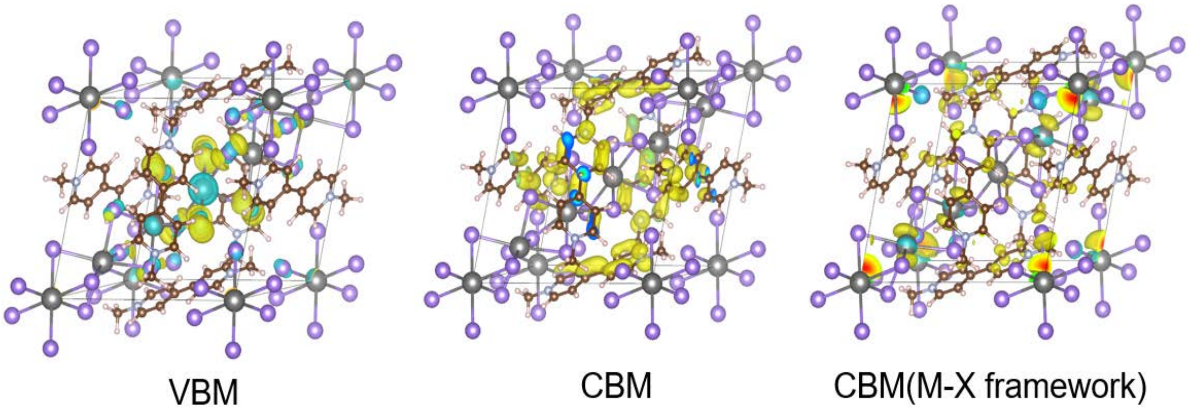

Supplementary Fig. 15 DFT results showing the CBM and VBM of 1D $MVPb_2I_6$

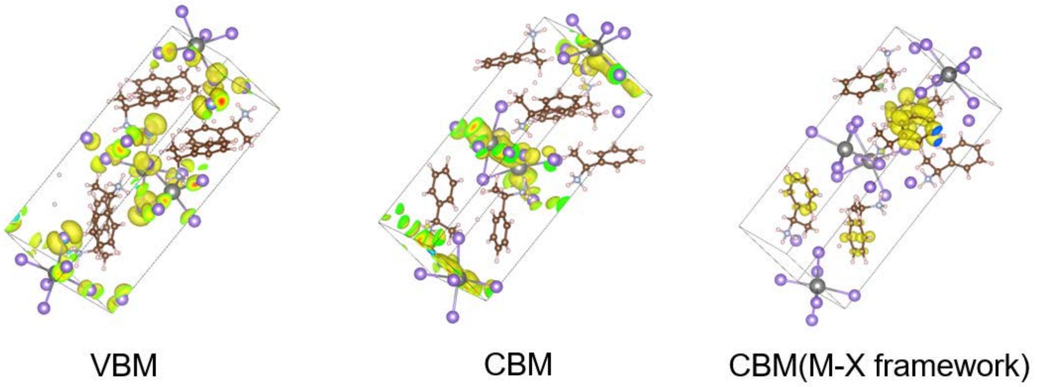

Supplementary Fig. 16 DFT results showing the CBM and VBM of 1D $MBAPbI_3$.

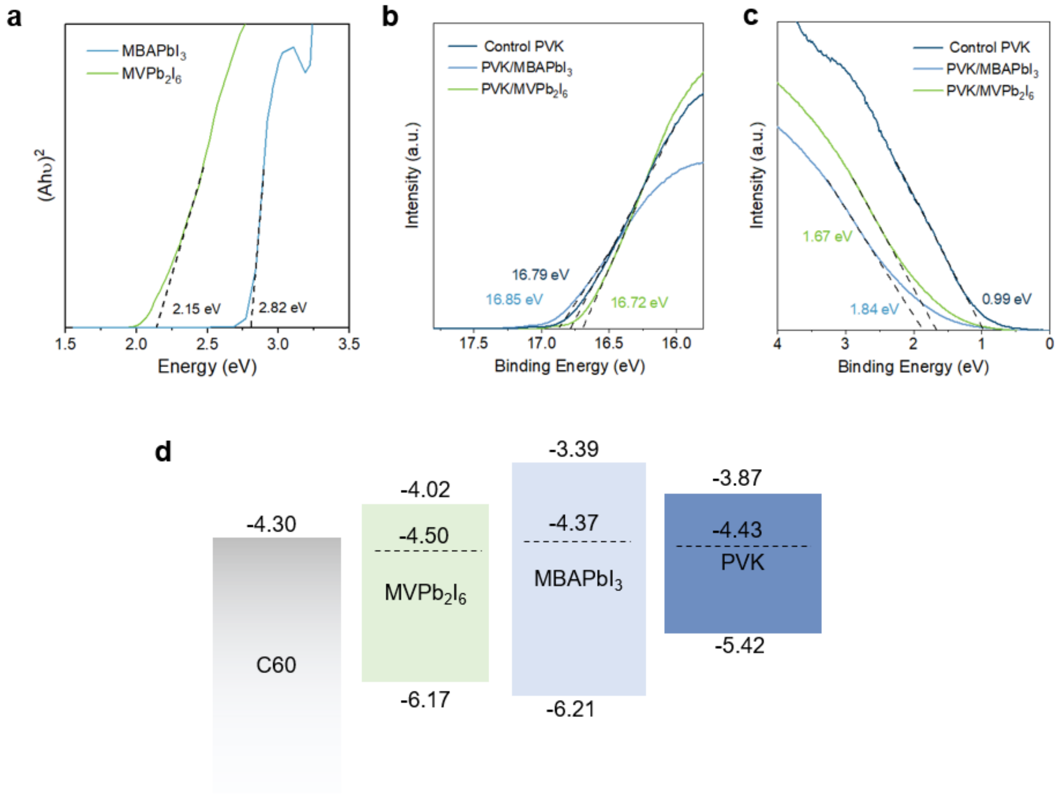

Supplementary Fig. 17 (**a**) Tauc plots of $MBAPbI_3$ and $MVPb_2I_6$, (**b**) and (**c**) UPS spectra of control perovskite (PVK), PVK/$MBAPbI_3$ and PVK/$MVPb_2I_6$ films, (**d**) Energy level diagram of ETL/perovskite interface.

Supplementary Table 1 Fitting results of TRPL of the ETL/perovskite structure for the control perovskite, perovskite/$MBAPbI_3$ and perovskite/$MVPb_2I_6$. Bi-exponential fitting method with the equation $y(t) = A_1exp(-t/\tau_1)+A_2exp(-t/\tau_2)+y_0$ was used. The average lifetime can be calculated with the equation $\tau_{avg} = (A_1\tau_1^2 + A_2\tau_2^2)/(A_1\tau_1 + A_2\tau_2)$.

Sample	A_1	τ_1 (ns)	A_2	τ_2 (ns)	τ_{avg} (ns)
Control PVK/C_{60}	0.81	18.80	0.53	25.67	22.04
PVK/$MBAPbI_3$/C_{60}	0.62	16.52	0.21	59.48	40.12
PVK/ $MVPb_2I_6$/C_{60}	0.68	0.96	0.60	13.02	12.09

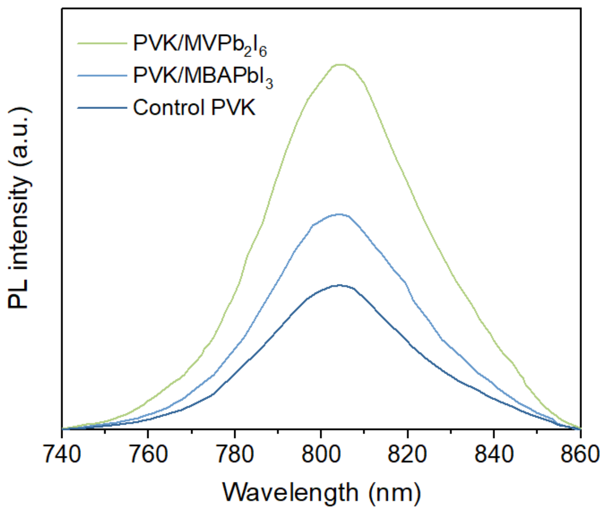

Supplementary Fig. 18 PL spectra of the control perovskite film, perovskite/$MBAPbI_3$ film and perovskite/$MVPb_2I_6$ film. The films are deposited on glass and the incident excitation light enters from the glass side.

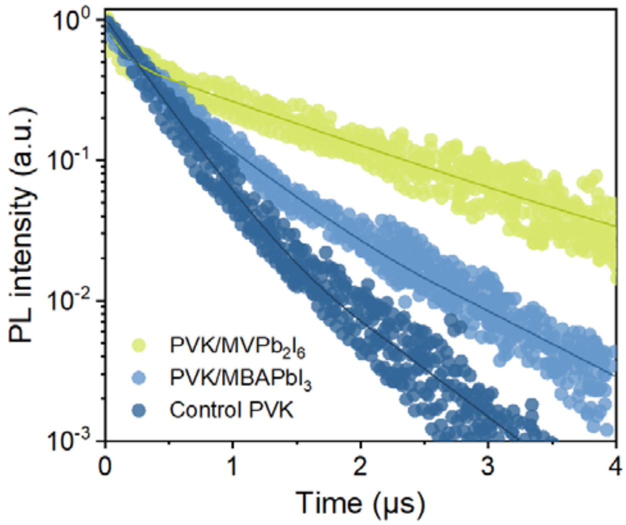

Supplementary Fig. 19 TRPL spectra of the control perovskite film, perovskite/$MBAPbI_3$ film and perovskite/$MVPb_2I_6$ film. The films are deposited on glass and the incident excitation light enters from the glass side.

Supplementary Table 2 Fitting results of the TRPL of the pristine perovskite films for the control perovskite, perovskite/$MBAPbI_3$ and perovskite/$MVPb_2I_6$. Bi-exponential fitting with the equation $y(t) = A_1exp(-t/\tau_1)+A_2exp(-t/\tau_2)+y_0$ was used. The average lifetime can be calculated with the equation $\tau_{avg} = (A_1\tau_1^2 + A_2\tau_2^2)/(A_1\tau_1 + A_2\tau_2)$.

Sample	A_1	τ_1 (ns)	A_2	τ_2 (ns)	τ_{avg} (ns)
Control PVK	0.36	317.46	0.61	353.73	341.17
PVK/$MBAPbI_3$	0.38	180.74	0.55	627.71	553.54
PVK/ $MVPb_2I_6$	0.42	101.62	0.53	1343.45	1273.22

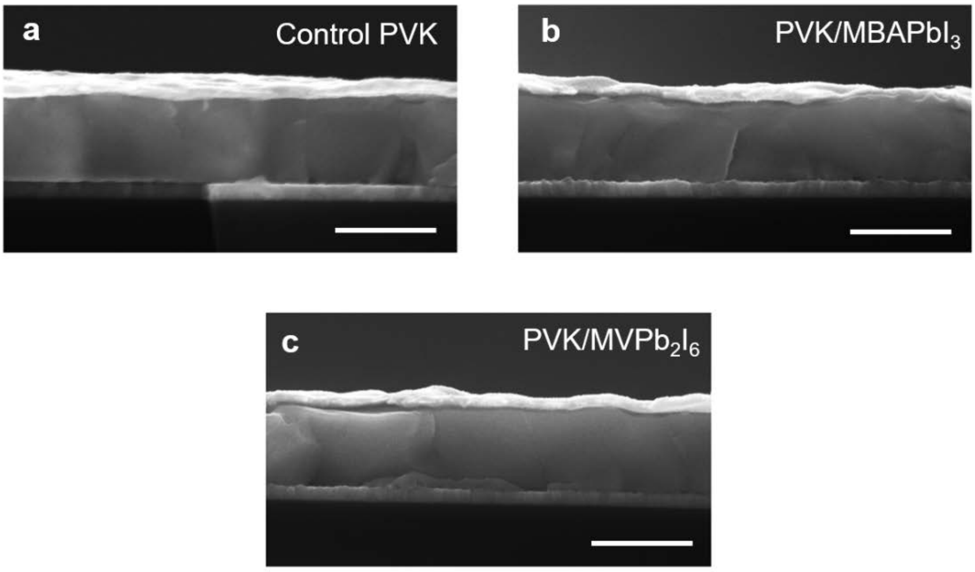

Supplementary Fig. 20 Cross-section SEM image of (**a**) control PVK, (**b**) PVK/$MBAPbI_3$, and (**c**) PVK/$MVPb_2I_6$ based devices. The scale bar is 1 μm.

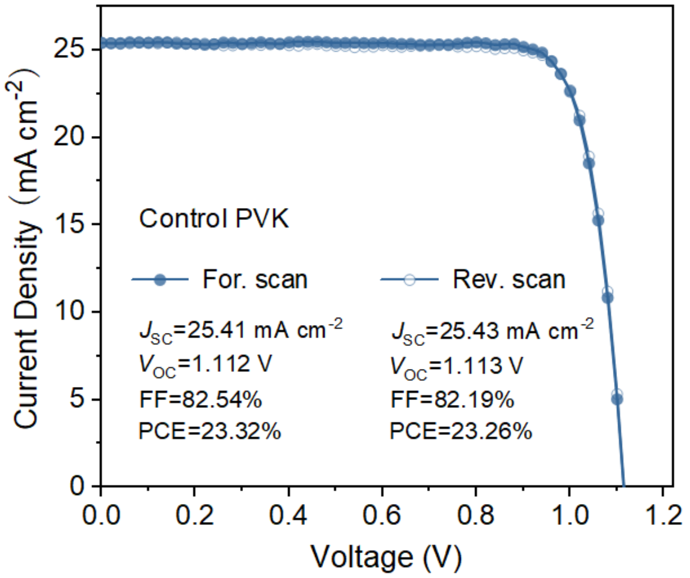

Supplementary Fig. 21 Forward and Reverse scan of the best performing devices based on control PVK.

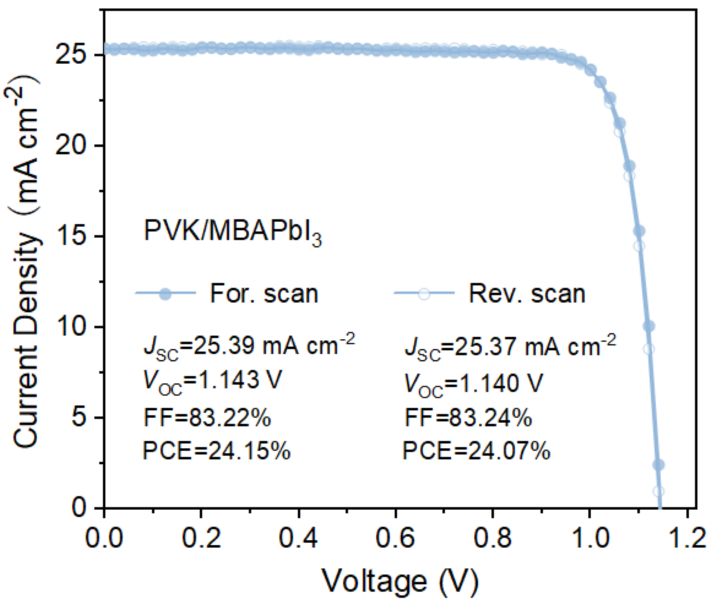

Supplementary Fig. 22 Forward and Reverse scan of the best performing devices based on PVK/$MBAPbI_3$.

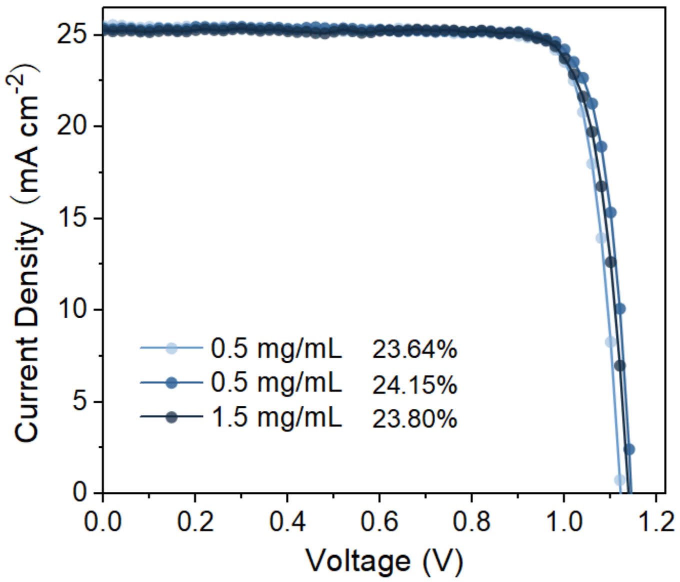

Supplementary Fig. 23 *J-V* curves of best-performing PSCs modified with different concentrations of MBAI.

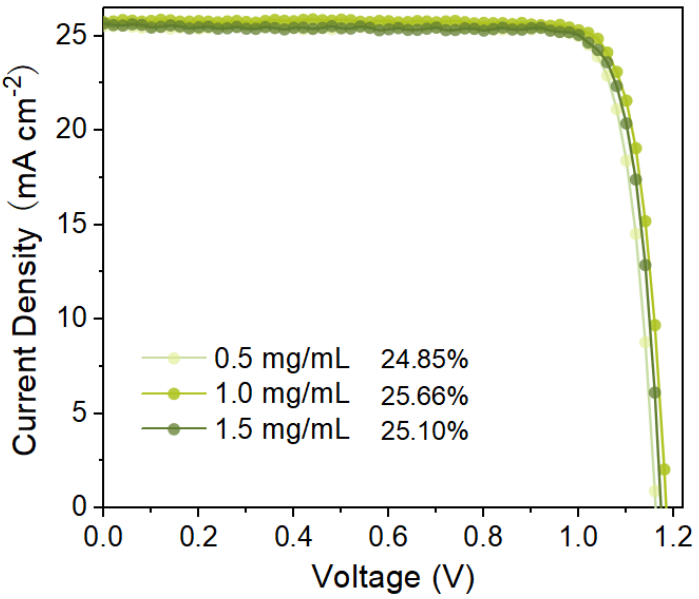

Supplementary Fig. 24 Photovoltaic parameters of best-performing PSCs modified with different concentrations of MVI_2.

Supplementary Table 3 Photovoltaic parameters of best-performing PSCs with different LD/3D perovskite.

Devices	V_{OC} (V)	J_{SC} (mA cm^{-2})	FF (%)	PCE (%)
Control-For.	1.112	25.41	82.54	23.32
Control-Rev.	1.113	25.43	82.19	23.26
PVK/$MBAPbI_3$-For.	1.143	25.39	83.22	24.15
PVK/$MBAPbI_3$-Rev.	1.140	25.37	83.24	24.07
PVK/$MVPb_2I_6$-For.	1.184	25.73	84.24	25.66
PVK/$MVPb_2I_6$-Rev.	1.185	25.65	84.13	25.57

Supplementary Table 4 Photovoltaic parameters of best-performing PSCs modified with different concentrations of MBA cation solution.

**Concen. (mg mL^{-1})**	**V_{OC} (V)**	**J_{SC} (mA cm^{-2})**	**FF (%)**	**PCE (%)**
0.5	1.120	25.50	82.78	23.64
1.0	1.143	25.39	83.22	24.15
1.5	1.137	25.27	82.83	23.80

Supplementary Table 5 Photovoltaic parameters of best-performing PSCs modified with different concentrations of MV cation solution.

**Concen. (mg mL^{-1})**	**V_{OC} (V)**	**J_{SC} (mA cm^{-2})**	**FF (%)**	**PCE (%)**
0.5	1.160	25.53	83.91	24.85
1.0	1.184	25.73	84.24	25.66
1.5	1.171	25.66	83.53	25.10

Supplementary Table 6 Statistics of state-of-the-art LD/3D heterojunctions for efficient PSCs.

**Strategies**	**Structure**	**Efficiency**	**Literatures**
Intact 2D/3D halide junction with $(BA)_2PbI_4$	*n-i-p*	24.63%	*Nat. Energy* 6, 63-71 (2021)
Grade 2D/3D heterojunction with BABr	*n-i-p*	23.78%	*Nat. Photonics* 15, 681-689 (2021)
3D/CLP/2D perovskite heterostructure with 4F-PEAI	*n-i-p*	21.2%	*Nat. Energy* 8, 294-303 (2023)
Phase purity modulation of 2D/3D heterojunction with BAI	*n-i-p*	24.5%	*Science* 377, 1425-1430 (2022)
2D/3D heterojunction with D-J $DMePDAI_2$	*n-i-p*	24.7%	*Science* 375, 71-76 (2022)
2D/3D heterojunction with $(Cl_4Tm)_2PbI_4$	*n-i-p*	24.6%	*Sci. Adv.* 9, eadg0032 (2022)
0D/3D heterojunction with m-$PBAI_2$	*n-i-p*	24.49%	*Sci. Adv.* 8, abk2722 (2022)
2D/3D heterojunction with RT treatment using OLAI molecule	*p-i-n*	24.3%	*Science* 376, 73-77 (2022)
2D/3D heterojunction with Quasi-2D treatment	*p-i-n*	23.3%	*Nat. Photonics* 16, 352-358 (2022)
2D/3D heterojunction with 2-aminoindan hydrochloride	*p-i-n*	25.12%	*Nat. Energy* 8 946-955 (2023)
0D/3D heterojunction with PEA_2ZnX_4	*p-i-n*	24.1%	*Nat. Energy* 8 284-293 (2023)
**1D/3D heterojunction with $MVPb_2I_6$**	***p-i-n***	**25.66%**	***This work***

中国认可
国际互认
检测
TESTING
CNAS L8490

Test and Calibration Center of New Energy Device and Module,
Shanghai Institute of Microsystem and Information Technology,
Chinese Academy of Sciences (SIMIT)

Measurement Report

Report No. 23TR100902

**Client Name**	Dr. Zhu Zonglong Research group
**Client Address**	83 Tat Chee Ave., City University of Hong Kong, Kowloon Tong, Hong Kong SAR
**Sample**	Perovskite Photovoltaic Cell
**Measurement Date**	9th October, 2023

**Performed by:**	Qiang Shi *Qiang Shi*	**Date:** 09/10/2023
**Reviewed by:**	Wenjie Zhao *Wenjie Zhao*	**Date:** 09/10/2023
**Approved by:**	Yucheng Liu *Yucheng Liu*	**Date:** 09/10/2023

Address: No.235 Chengbei Road, Jiading, Shanghai **Post Code:**201800
E-mail: solarcell@mail.sim.ac.cn **Tel:** +86-021-69976921

The measurement report without signature and seal are not valid.
This report shall not be reproduced, except in full, without the approval of SIMIT.

Sample Information

Sample Type	Perovskite solar cell
Serial No.	222-1#
Lab Internal No.	23100902-1#
Measurement Item	I-V characteristic
Measurement Environment	24.4±2.0°C,43.6±5.0%R.H

Measurement of I-V characteristic

**Reference cell**	PVM 1121
**Reference cell Type**	mono-Si, WPVS, calibrated by NREL (Certificate No. ISO 2075)
**Calibration Value/Date of Calibration for Reference cell**	144.53mA/ Feb. 2023
**Measurement Conditions**	Standard Test Condition (STC):<br>Spectral Distribution: AM1.5 according to IEC 60904-3 Ed.3,<br>Irradiance: 1000±50W/m^2, Temperature: 25±2°C
**Measurement Equipment/ Date of Calibration**	AAA Steady State Solar Simulator (YSS-T155-2M) / July.2023<br>IV test system (ADCMT 6246) / June. 2023<br>SR Measurement system (CEP-25ML-CAS) / April.2023<br>Measuring Microscope (MF-B2017C) / July.2023
**Measurement Method**	I-V Measurement:<br>Linear sweep in direct direction based on IEC 60904-1:2020;<br>Spectral Mismatch factor was calculated according to IEC 60904-7 and I-V correction according to IEC 60891;
**Measurement Uncertainty**	Area: 1.0%(k=2); Isc: 1.9%(k=2); Voc: 1.0%(k=2);<br>Pmax: 2.4%(k=2); Eff: 2.5%(k=2)

Report No. 23TR100902

====Measurement Results ====

	Forward Scan (Isc to Voc)	Reverse Scan (Voc to Isc)
Area	5.08 mm^2	
Isc	1.302 mA	1.303 mA
Voc	1.179 V	1.179 V
Pmax	1.257 mW	1.280 mW
Ipm	1.219 mA	1.239 mA
Vpm	1.031 V	1.033 V
FF	81.85 %	83.32 %
Eff	24.75 %	25.20 %

- Spectral Mismatch Factor: SMM=0.9902.
- Designated illumination area defined by a thin mask was measured by measuring microscope.
- Test results listed in this measurement report refer exclusively to the mentioned measured sample.
- The results apply only at the time of the test, and do not imply future performance.

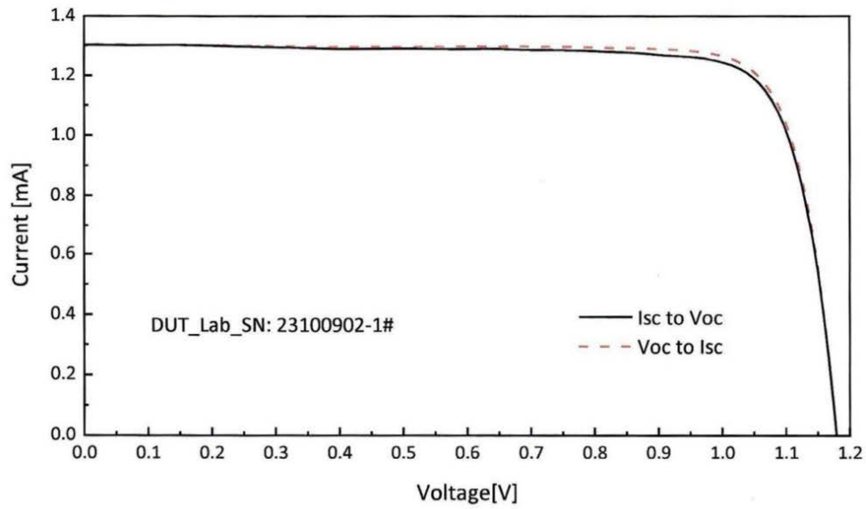

Fig.1 I-V curves of the measured sample

3 / 4

Supplementary Fig. 25 Independent efficiency certification of PVK/$MVPb_2I_6$ based solar cells by an accredited institute of Shanghai Institute of Microsystem and Information Technology, Chinese Academy of Sciences (SIMIT). Logos shown in this report are reproduced with permission from SIMIT.

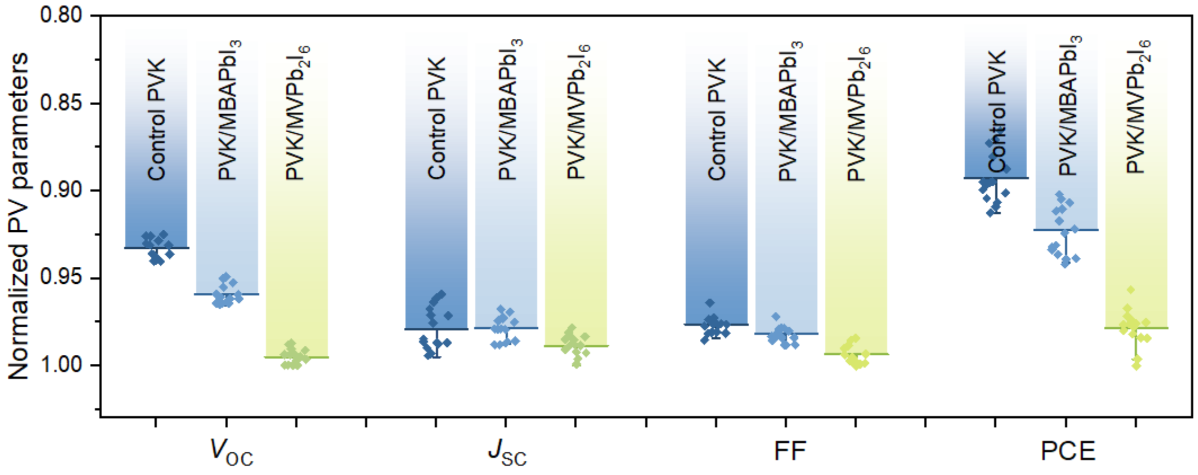

Supplementary Fig. 26 Photovoltaic parameters of 15 individual devices with different heterostructures. The scatter points represent the parameters of each device, and the histogram is the average value. Data are normalized to the highest value of each parameter.

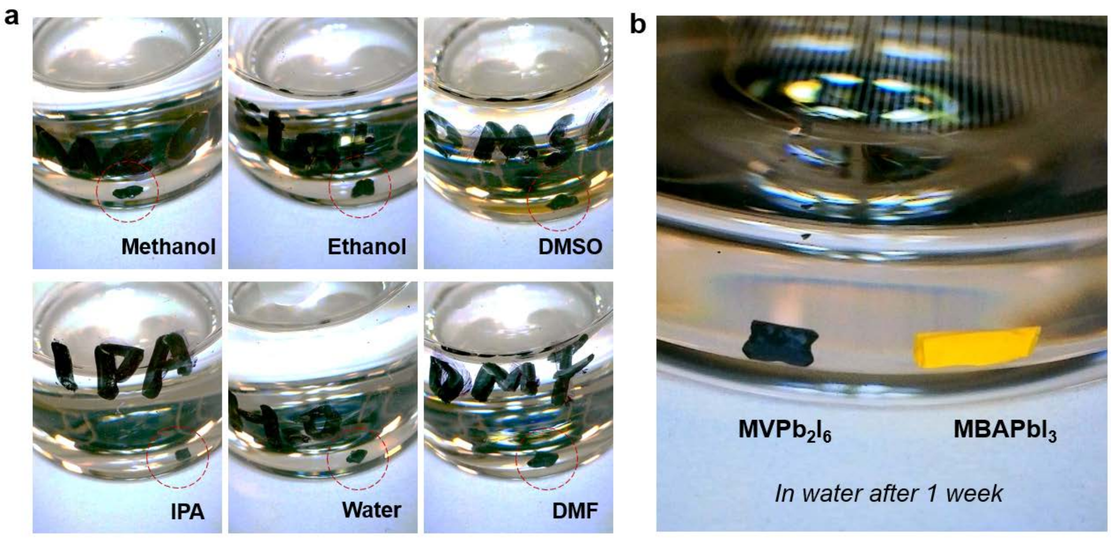

Supplementary Fig. 27 (**a**) Photos of the stability of the $MVPb_2I_6$ single crystals measured in different polar solvent and (**b**) comparison with $MBAPbI_3$ single crystals stored in water for 1 week.

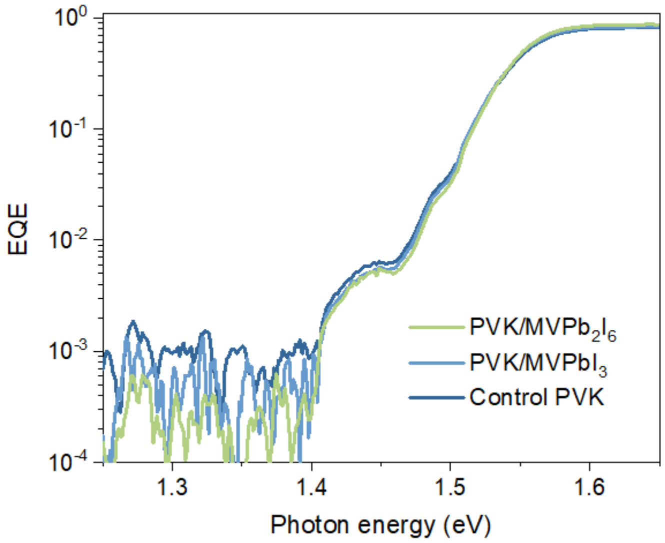

Supplementary Fig. 28 Sensitive-EQE curves of PSCs based on the control, $PVK/MBAPbI_3$ and $PVK/MVPb_2I_6$.

Supplementary Note 2

The detailed $V_{\mathrm{OC,loss}}$ analysis can be described by the equation listed below:

$$q\Delta V = E_{\mathrm{g}} - qV_{\mathrm{OC}}$$

$$= \left(E_{\mathrm{g}} - qV_{\mathrm{OC}}^{\mathrm{SQ}}\right) + \left(qV_{\mathrm{OC}}^{\mathrm{SQ}} - qV_{\mathrm{OC}}^{\mathrm{rad}}\right) + \left(qV_{\mathrm{OC}}^{\mathrm{rad}} - qV_{\mathrm{OC}}\right)$$

$$= \left(E_{g} - qV_{\mathrm{OC}}^{\mathrm{SQ}} + q\Delta \mathrm{V}_{\mathrm{OC}}^{\mathrm{SQ}}\right) + \Delta qV_{\mathrm{OC}}^{\mathrm{rad}} + \Delta qV_{\mathrm{OC}}^{\mathrm{non-rad}}$$

$$= q(\Delta V_{1} + \Delta V_{2} + \Delta V_{3}) \cdots \text{ (S3)}$$

where q, ΔV, E_{g} is the elementary charge, the total voltage loss, and the bandgap of perovskite, respectively. $V_{\mathrm{OC}}^{\mathrm{SQ}}$ is the S-Q limit of open circuit voltage, $V_{\mathrm{OC}}^{\mathrm{rad}}$ is the V_{OC} without non-radiative recombination occurring in PSCs, $\Delta V_{\mathrm{OC}}^{\mathrm{SQ}}$ is the V_{OC} loss due to the non-ideal EQE above the bandgap, $\Delta V_{\mathrm{OC}}^{\mathrm{rad}}$ is the $\mathrm{V_{OC}}$ loss due to the sub-bandgap radiative recombination, and $\Delta V_{\mathrm{OC}}^{\mathrm{non\text{-}rad}}$ is the $\mathrm{V_{OC}}$ loss of non-radiative recombination. The calculation method was following detailed balance theory. Therefore, the energy loss can be divided into three parts, ΔV_1, ΔV_2 and ΔV_3, which represent radiative recombination above E_{g}, energy loss from blackbody radiation and voltage loss induced by the nonradiative recombination, respectively.

Supplementary Table 7 Results of calculated V_{OC} loss in the control PSCs and LD/3D PSCs.

**Device**	**$E_{g,PV}$ (eV)**	**$V_{OC,SQ}$ (V)**	**V_{OC} (V)**	**ΔV_1 (mV)**	**ΔV_2 (mV)**	**ΔV_3 (mV)**	**$V_{OC,loss}$ (mV)**	**V_{OC}^* (V)**
Control	1.548	1.276	1.112	273.91	84.41	88.98	447.31	1.101
MBA	1.548	1.276	1.143	273.91	60.33	75.45	409.70	1.138
MV	1.548	1.276	1.185	273.59	29.86	64.65	368.11	1.180
V_{OC} is the value extracted from *J-V* curve								
V_{OC}^* is the value based on the $E_{g,PV}$ and $V_{OC,loss}$								

Supplementary Note 3

For light intensity dependent open-circuit voltage measurements, the ideality factor (n) can be extracted according to $V_{OC}(P) = n\mathbf{k}T/q \cdot \ln(P) + C$, where T is the absolute temperature, P is the incident light intensity, q is the elementary charge, C is a constant, **k** is the Boltzmann constant, and T is the absolute temperature. In general, an ideality factor of 1 is associated to bimolecular bond-to-bond radiative recombination of carriers or dominating Shockley–Read–Hall (SRH) trap-assisted recombination with one pinned charge carrier density, while an ideality factor of 2 is associated with dominated SRH recombination without pinning of one charge carrier density.

Supplementary Note 4

For FF loss analysis, the FF losses in high-performing PSCs are determined by two main factors, non-radiative loss and charge transport loss. The maximum FF (FF_{max}) can be empirically calculated according to the following equation:

$$FF_{\max} = \frac{\nu_{\mathrm{OC}} - \ln(\nu_{\mathrm{OC}} + 0.72)}{\nu_{\mathrm{OC}} + 1} \cdots \text{(S4)}$$

where $\nu_{OC} = \frac{V_{OC}}{nK_{\mathbf{B}}T/q}$ (n is ideality factor, $K_{\mathbf{B}}$ is Boltzmann constant, T is temperature, q is elementary charge). The ideality factors were extracted from the V_{OC} as a function of light intensity on a logarithmic scale, which were shown in Fig. 4g.

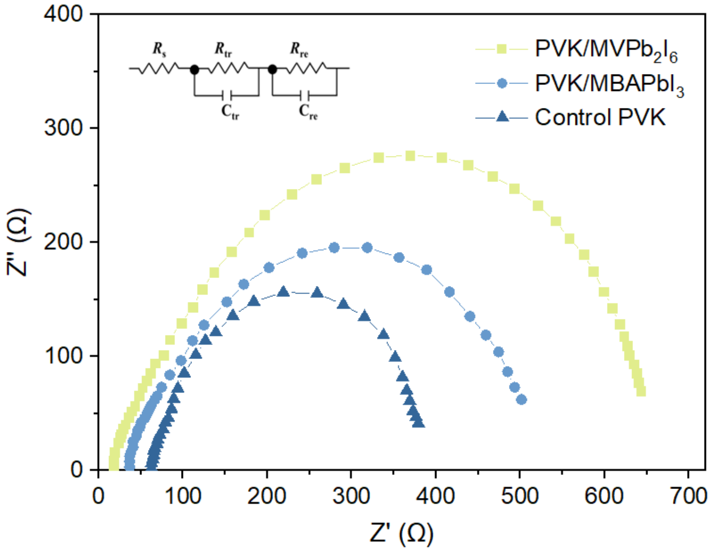

Supplementary Fig. 29 EIS spectra of PSCs based on the control the control, PVK/$MBAPbI_3$ and PVK/$MVPb_2I_6$.
