# Supplementary material for: Harnessing strong aromatic conjugation in low-dimensional perovskite heterojunctions for high-performance photovoltaic devices
Source: Nat Commun. 2024 Mar 29;15:2753. doi: 10.1038/s41467-024-47112-y (PMC10980693; doi:10.1038/s41467-024-47112-y)
Supplement: Supplementary file 3 — Reporting Summary [file 41467_2024_47112_MOESM3_ESM.pdf]

nature portfolio

Corresponding author(s): Haipeng Lu, Xiao Cheng Zeng, Zonglong Zhu

Solar Cells Reporting Summary

Nature Portfolio wishes to improve the reproducibility of the work that we publish. This form is intended for publication with all accepted papers reporting the characterization of photovoltaic devices and provides structure for consistency and transparency in reporting. Some list items might not apply to an individual manuscript, but all fields must be completed for clarity.

For further information on Nature Research policies, including our data availability policy, see Authors & Referees.

Experimental design

Please check the following details are reported in the manuscript, and provide a brief description or explanation where applicable.

1. Dimensions

Area of the tested solar cells

- [x] Yes: 0.0414 cm2
- [ ] No: *Explain why this information is not reported/not relevant.*

Method used to determine the device area

- [x] Yes: See Page 32-34 in Supplementary Information.
- [ ] No: *Explain why this information is not reported/not relevant.*

2. Current-voltage characterization

Current density-voltage (J-V) plots in both forward and backward direction

- [x] Yes: See Figure 3 in Manuscript and Fig. 21 and 22 in Supplementary Information.
- [ ] No

Voltage scan conditions

- [x] Yes: J-V curves were acquired using a Keithley 2400 source meter under a reverse scan sweep mode (from 1.20 V to -0.01 V) and a forward scan sweep mode (from -0.01 V to 1.20 V), with a scan rate of 0.01 V s-1 and a delay time of 10 ms.
- [ ] No: *Explain why this information is not reported/not relevant.*

Test environment

- [x] Yes: In N2 at room temperature.
- [ ] No: *Explain why this information is not reported/not relevant.*

Protocol for preconditioning of the device before its characterization

- [ ] Yes: *Provide a description of the protocol.*
- [x] No: No precondition was needed before measurement.

Stability of the J-V characteristic

- [x] Yes: See Figure 3e in Manuscript.
- [ ] No: *Explain why this information is not reported/not relevant.*

3. Hysteresis or any other unusual behaviour

Description of the unusual behaviour observed during the characterization

- [ ] Yes: *Provide a description of hysteresis or any other unusual behaviour observed during the characterization.*
- [x] No: No unusual behaviour

Related experimental data

- [x] Yes: See Figure 3b in Manuscript.
- [ ] No: *Explain why this information is not reported/not relevant.*

4. Efficiency

External quantum efficiency (EQE) or incident photons to current efficiency (IPCE)

- [x] Yes: See Figure 3c in Manuscript. External quantum efficiency (EQE) measurements were performed using a QE-R EQE system
- [ ] No: *Explain why this information is not reported/not relevant.*

A comparison between the integrated response under the standard reference spectrum and the response measure under the simulator

- [x] Yes: See Figure 3c in Manuscript.
- [ ] No: *Explain why this information is not reported/not relevant.*

April 2023

For tandem solar cells, the bias illumination and bias voltage used for each subcell

- [ ] Yes — *Provide a description of the measurement conditions.*
- [x] No — Not Tandem solar cells.

5. Calibration

Light source and reference cell or sensor used for the characterization

- [x] Yes — The light intensity was calibrated to 100 mW cm-2 using a silicon reference cell with a KG2 filter.
- [ ] No — *Explain why this information is not reported/not relevant.*

Confirmation that the reference cell was calibrated and certified

- [x] Yes — SIMIT
- [ ] No — *Explain why this information is not reported/not relevant.*

Calculation of spectral mismatch between the reference cell and the devices under test

- [ ] Yes — *Provide a value of the spectral mismatch and/or a description of how it has been taken into account in the measurements.*
- [x] No — No spectral mismatch

6. Mask/aperture

Size of the mask/aperture used during testing

- [x] Yes — 0.0414 cm2
- [ ] No — *Explain why this information is not reported/not relevant.*

Variation of the measured short-circuit current density with the mask/aperture area

- [ ] Yes — *Report the difference in the short-circuit current density values measured with the mask and aperture area.*
- [x] No — N/A

7. Performance certification

Identity of the independent certification laboratory that confirmed the photovoltaic performance

- [x] Yes — SIMIT
- [ ] No — *Explain why this information is not reported/not relevant.*

A copy of any certificate(s)

- [x] Yes — See Page 32-34 in Supplementary Information.
- [ ] No — *Explain why this information is not reported/not relevant.*

8. Statistics

Number of solar cells tested

- [x] Yes — 15 individual devices
- [ ] No — *Explain why this information is not reported/not relevant.*

Statistical analysis of the device performance

- [x] Yes — See Figure 3d in Manuscript and Fig. 26 in Supplementary Information.
- [ ] No — *Explain why this information is not reported/not relevant.*

9. Long-term stability analysis

Type of analysis, bias conditions and environmental conditions

- [x] Yes — The ISOS-L-1I stability was conducted by applying the perovskite solar cells under a 1 sun equivalent LED lamp in a N2-filled glovebox (with the contents of O2 and H2O <10 ppm) at room temperature. The PSCs were biased at maximum-power-point (MPP) voltage and the power output was tracked by using a multi-potentiostat (CHI1040C, CH Instruments, Inc.). During the MPP test, the current density-voltage (J-V) curves of the devices were obtained every 12 h to get the proper loads for the MPP. The ISOS-D-1 stability was conducted to study the evolution of normalized PCE for non-encapsulated solar cells aged at room temperature of RH of 20-40% in the dark.
- [ ] No — *Explain why this information is not reported/not relevant.*
